# Genetic diversity and drug resistance among newly diagnosed and antiretroviral treatment-naive HIV-infected individuals in western Yunnan: a hot area of viral recombination in China

**DOI:** 10.1186/1471-2334-12-382

**Published:** 2012-12-28

**Authors:** Min Chen, Yanling Ma, Song Duan, Hui Xing, Shitang Yao, Yingzhen Su, Hongbing Luo, Li Yang, Huichao Chen, Liru Fu, Aijuan Qu, Chin-Yih Ou, Manhong Jia, Lin Lu

**Affiliations:** 1 Yunnan Center for AIDS/STD Control and Prevention, Yunnan Center for Disease Control and Prevention, Kunming, Yunnan, 650022, China; 2 Department of HIV/AIDS Control and Prevention, Dehong Center for Disease Control and Prevention, Dehong, Yunnan, 678400, China; 3 National Center for AIDS/STD Control and Prevention, Chinese Center for Disease Control and Prevention, Beijing, 102206, China; 4 Laboratory of Metabolism, Center for Cancer Research National Cancer Institute, National Institutes of Health, Bethesda, MD, 20892, USA; 5 Global AIDS Program in China, U. S. Centers for Disease Control and Prevention, Beijing, 100600, China

**Keywords:** HIV-1, Genetic diversity, Drug resistance, Injecting drug use, Dehong, China

## Abstract

**Background:**

The emergence of an HIV-1 epidemic in China was first recognized in Dehong, western Yunnan. Due to its geographic location, Dehong contributed greatly in bridging HIV-1 epidemics in Southeast Asia and China through drug trafficking and injection drug use; and also extensively to the HIV genetic diversity in Yunnan and China. We attempt to monitor HIV-1 in this area by studying the HIV-1 genetic distribution and transmitted drug resistance (TDR) in various at-risk populations.

**Methods:**

Blood samples from a total of 320 newly HIV-1 diagnosed individuals, who were antiretroviral therapy (ART)-naive, were collected from January 2009 to December 2010 in 2 counties in Dehong. HIV-1 subtypes and *pol* gene drug resistance (DR) mutations were genotyped.

**Results:**

Among 299 *pol* sequences successfully genotyped (93.4%), subtype C accounted for 43.1% (n=129), unique recombinant forms (URFs) for 18.4% (n=55), CRF01_AE for 17.7% (n=54), B for 10.7% (n=32), CRF08_BC for 8.4% (n=25) and CRF07_BC for 1.7% (n=5). Subtype distribution in patients infected by different transmission routes varied. In contract to the previous finding of CRF01_AE predominance in 2002-2006, subtype C predominated in both injecting drug users (IDUs) and heterosexually transmitted populations in this study. Furthermore, we found a high level of BC, CRF01_AE/C and CRF01_AE/B/C recombinants suggesting the presence of active viral recombination in the area. TDR associated mutations were identified in 4.3% (n=13) individuals. A total of 1.3% of DR were related to protease inhibitors (PIs), including I85IV, M46I and L90M; 0.3% to nucleoside reverse transcriptase inhibitors (NRTIs), including M184I; and 2.7% to non-nucleoside reverse transcriptase inhibitors (NNRTIs), including K103N/S, Y181C, K101E and G190A.

**Conclusion:**

Our work revealed diverse HIV-1 subtype distributions and intersubtype recombinations. We also identified a low but significant TDR mutation rate among ART-naive patients. These findings enhance our understanding of HIV-1 evolution and are valuable for the development and implementation of a comprehensive public health approach to HIV-1 DR prevention and treatment in the region.

## Background

Dehong prefecture is located in western Yunnan bordering northern Myanmar. It is in a key position along the drug trafficking routes from Southeast Asia’s opium-producing and high HIV prevalent “Golden Triangle” region into China [1]. The first HIV epidemic in China with the identification of 146 infected IDUs was reported in Dehong in 1989 [2]. To date, Dehong remains one of the most HIV-affected areas in China [3,4].

Subtype B and subtype B' (the B subtype originally found in Thailand) were the HIV-1 strains first found among infected Dehong IDUs [5-7]. The proportion of subtype B' among the subtype B category increased from 20% in 1990 to 90% in 1996 [5-7]. Subtype C was identified in IDUs from India in the early 1990s [8] and cocirculated with B' in the IDUs in western Yunnan in the first half of the 1990s resulting in many recombinant viruses [9-12]. Another CRF01_AE subtype was also found in this region in 1994 [13] and, with increased sexual contacts, became the predominant strain in 2000 [9,10]. Other viral strains, CRF07_BC and CRF08_BC, were also found in Dehong in 2002 [9,10,12].

To combat the growing HIV infection, China initiated a national free ART program with standardized treatment options and management protocols in 2003 and by the end of 2010, the total number of ART patients exceeded 100,000. Although the widespread use of highly active ART successfully decreases HIV/AIDS-related mortality and morbidity [14,15], suboptimal ART adherence may accelerate the emergence of drug resistant viruses [16] and compromise ART efficacy in treated individuals and transmission recipients. It was reported that suboptimal ART management in central China resulted in the rise of DR rate from 13.9%, 45.4% to 62.7% in individuals who were ART-naive and 3- and 6-months post-ART, respectively [17]. By the end of 2010, of the 19,512 ART patients in Yunnan, 20% resided in Dehong. It is important to conduct HIV DR surveys to estimate the TDR rate among ART-naive populations with different transmission routes to provide baseline information for the formulation of effective ART regimens to delay the emergence of DR and to develop a rational public health strategy to control the HIV epidemic.

## Methods

### Study area, population and sampling

Blood samples were collected from 320 individuals newly diagnosed with HIV-1 infection in Longchuang and Luxi counties of Dehong prefecture between January 2009 and December 2010. HIV-1 infection was confirmed by Western Blot assay (HIV BLOT 2.2, MP Diagnostics, Singapore). All study participants were ART-naive and provided informed consent. This study was approved by the institutional review board of Yunnan Center for Disease Control and Prevention.

### Amplification and sequencing of HIV-1 *pol*

Viral RNA was extracted from 200 μl plasma using an automated extractor (MagNA Pure LC system, Roche, Branchburg, NJ) according to the manufacturer’s instructions. For the amplification of the *pol* regions, an in-house nested reverse transcription PCR method was used. The target sequence was amplified with One Step Reverse Transcription PCR reagents procured from Takara (Dalian, China) using primers MAW26 (5^′^-TTGGAAATGTGGAAAGGAAGGAC-3^′^) and RT21 (5^′^-CTGTATTTCTG CTATTAAGTCTTTTGATGGG-3^′^) in a 25 μl reaction for 30 cycles. Cycling conditions were 50°C for 30 min, 94°C for 5 min, 94°C for 30 s,55°C for 30 s,72°C for 2.5 min, followed with an extension at 72°C for 10 min. The nested PCR was performed using Taq PCR master mix (Tiangen, Beijing, China), using primers PRO-1 (5^′^-CAGAGCCAACAGCCCCACCA-3^′^) and RT20 (5^′^-CTGCCAGTTCTAGCTCTGCTTC-3^′^) in a 50 μl reaction for 30 cycles and the cycling conditions were 94°C for 5 min, 94°C for 30 s, 63°C for 30 s, 72°C 2.5 min, followed with an extension at 72°C for 10 min. The resulting PCR product of 1191 bp in length contained a full length protease (PR) gene of 99 amino acid codons and the first 298-codon segment of the reverse transcriptase (RT) gene. PCR products were visualized by 1% agarose gel electrophoresis. Successfully amplified samples were sequenced by Biomed Co. (Beijing, China) on an ABI 3730xl automated DNA analyzer (Applied Biosystems, Foster City, CA) with primers: PROS3 (5^′^-GCCAACAGCCCCACCA-3^′^), RTAS (5^′^-CTCAGATTGGTTGCAC-3^′^), RTB (5^′^-CCTAGTATAAACAATGAGACAC-3^′^), PROC1S (5^′^-GCTGGGTGTGGTATTCC-3^′^) and RT20S3 (5^′^-GTTCTAGCTCTGCTTC-3^′^). Each step was carried out with appropriate negative controls to detect PCR-related contamination during the experiments.

### Sequence analysis

The sequence contig assembly was performed using analysis software, Sequencher 4.9 (Gene Codes Corporation, Ann Arbor, MI). The ClustalW Multiple alignment and manual editing were performed using BioEdit Sequence Alignment Editor (Ibis Biosciences, Carlsbad, CA). Edited sequences were evaluated for DR mutations using both the Surveillance Drug Resistance Mutations (SDRM) list recommended by the World Health Organization [18], and the Stanford University Algorithm (http://hivdb.stanford.edu). The HIV-1 subtypes of *pol* sequences were identified by the REGA HIV subtyping tool (http://www.bioafrica.net/subtypetool/html/) [19]. To demonstrate possible intersubtype mosaicism, candidate sequences were analyzed using the Recombination Identification Program (RIP; version 3.5.1) available at the HIV sequence database site (http://hiv-web.lanl.gov). Bootscanning analysis was performed with default parameters of window: 200bp, step: 20bp, gapsrip: on, reps: 100, Kinura (2-parameter), T/t: 2.0. Similarity plot analysis (SimPlot version 3.5.1; S. Ray, Johns Hopkins University, Baltimore, MD; http://sray.med.som.jhmi.edu/RaySoft/SimPlot/) was performed using reference A1, B, C, CRF07_BC, CRF08_BC and CRF01_AE strains. All sequences obtained in this study were submitted to GenBank with accession numbers from JQ658474 to JQ658772.

### Statistical analysis

Statistical analysis was conducted using the SPSS 17.0 package (SPSS Inc. Chicago, IL). Categorical variables were compared using chi-square analysis. When the theoretical frequency was less than 5, Fisher exact test was used. All tests were two-tailed and a *p*-value <0.05 was considered significant.

## Results

### Demographic characteristics of patients

A total of 320 individuals diagnosed with HIV-1 infection between 2009 and 2010 were recruited from two counties in Dehong prefecture. Two hundred ninety nine sequences (93.4%) of HIV-1 *pol* region were successfully amplified and sequenced. The demographics of these individuals are shown in Table 1. The ratio of males to females was 1:0.78 and the mean age was 32.5 years (range: 2-74 years). Heterosexual transmission constituted the most frequent transmission route (75.9%, n=227), followed by intravenous drug injection (17.4%, n=52), mother-to-infant transmission (1.3%, n=4) and homosexual transmission (0.7%, n=2). The transmission route of the remaining 4.7% (n=14) individuals was unknown. The ethnicities of the studied individuals were Han (40.8%, n=122), Jingpo (32.8%, n=98), Dai (21.4%, n=64), Achang (2.3%, n=7), Deang (1.3%, n=4), Bai (1.0%, n=3), and Dongxiang (0.3%, n=1). There were also 10 Burmese individuals. In addition, 28.4% (n=85) of the individuals had CD4 cell count lower than 350 cells/μl and were ART-eligible.

**Table 1:** Demographic characteristics and HIV DR of study individuals

	** Total N (%)**		**No. of resistant mutations**	**Resistance rate (%)**
**Total**	299	(100)	13	4.5
**Gender**				
Male	168	(56.2)	7	4.3
Female	131	(43.8)	6	4.8
**Age**				
≤25	75	(25.1)	3	4.2
26-35	130	(43.5)	6	4.8
36-45	58	(19.4)	4	7.4
>45	36	(12.0)	0	0
**Transmission routes**				
Heterosexual	227	(75.9)	8	3.7
Intravenous drug injection	52	(17.4)	5	10.6
Homosexual	2	(0.7)	0	0
Mother to child	4	(1.3)	0	0
Unknown	14	(4.7)	0	0
**Ethnicity**				
Han	122	(40.8)	3	2.5
Jingpo	98	(32.8)	7	7.7
Dai	64	(21.4)	2	3.2
Others	15	(5.0)	1	7.1
**CD4**^**+**^**Count** (cells/μl)				
≤200	21	(7.0)	1	5.0
201-350	64	(21.4)	3	4.9
351-500	88	(29.4)	4	4.8
≥501	126	(42.1)	5	4.1
**HIV-1 Subtype**				
C	129	(43.1)	7	5.7
URFs	55	(18.4)	2	3.8
CRF01_AE	53	(17.7)	1	1.9
B	32	(10.7)	2	6.7
CRF08_BC	25	(8.4)	1	4.2
CRF07_BC	5	(1.7)	0	0.0

Abbriviations: URFs, unique recombinant forms; CRFs, circulating recombinant forms.

### HIV-1 subtypes distribution

**Figure 1:**
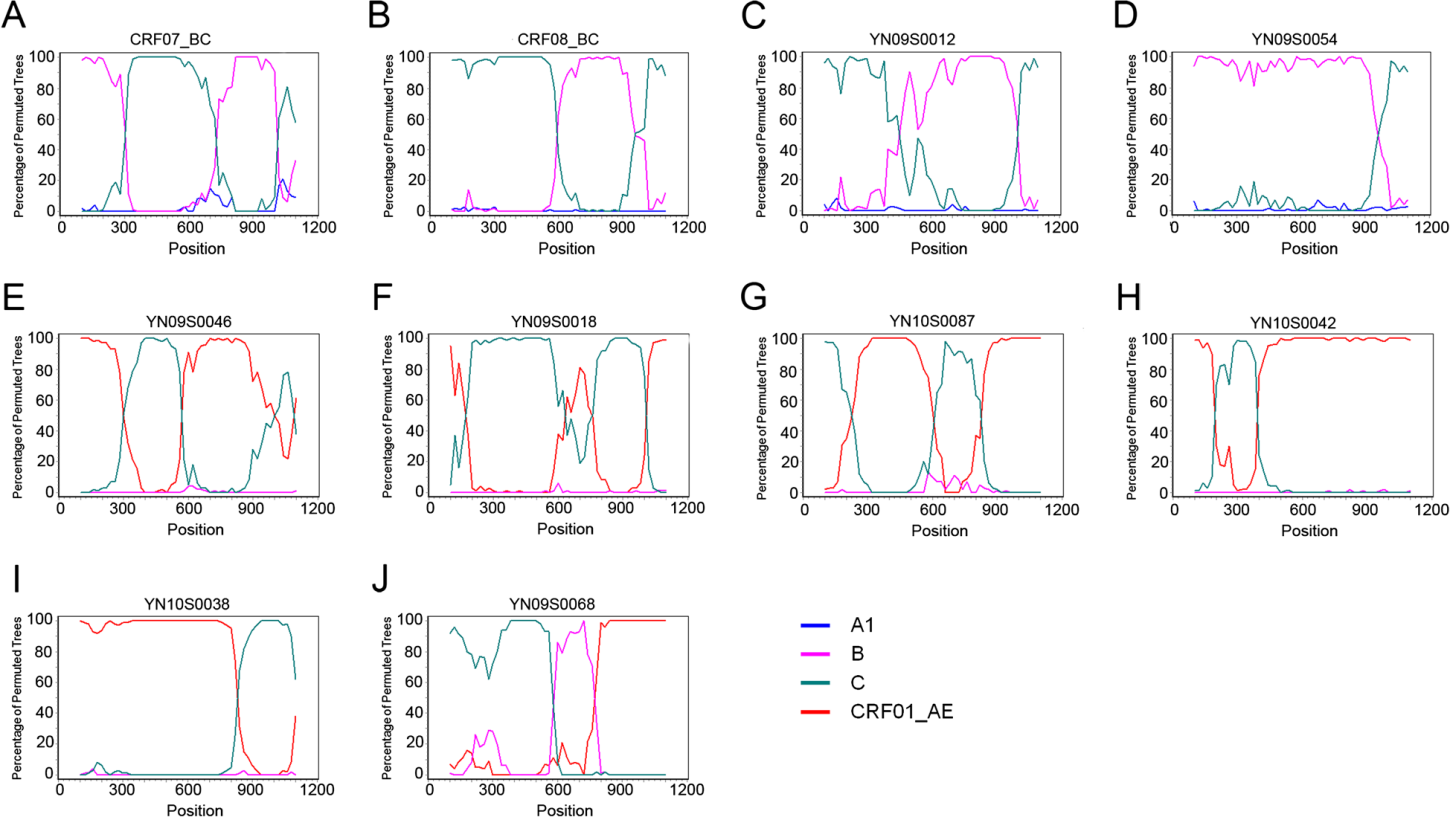
**Bootscanning analysis of new viral recombination in HIV-1 *pol *gene region.** (**A**) the reference sequence of CRF07_BC; (**B**) the reference sequence of CRF08_BC; (**C**) representative BC recombinant YN09S0012; (**D**) representative BC recombinant, YN09S0054; (**E** to **I**), representative CRF_01AE/C recombinant, **E:** YN09S0046, **F:** YN10S0018, **G:** YN10S0087, **H:** YN10S0042 and **I:** YN10S0038; and (**J**) representative CRF_01AE/B/C recombinant, YN09S0068. The conditions used for this analysis were following: Window: 200 bp, step: 20 bp, gapsrip: on, reps: 100, Kinura (2-parameter), T/t: 2.0.

HIV-1 *pol* gene sequences (1191 bp) were subjected to subtype determination [20]. As revealed in Table 1, 6 basic subtypes were identified. HIV-1 C subtype was found to be the most common subtype (43.1%, n=129), followed by URFs (18.4%, n=55), CRF01_AE (17.7%, n=53), B (10.7%, n=32), CRF08_BC (8.4%, n=25) and CRF07_BC (1.7%, n=5). Bootscanning analysis revealed the presence of 55 (18.4%) URFs including 48 BC recombinants (16.1%, Figure 1C and D), 6 CRF_01AE/C (2.0%, Figure 1E-I) and 1 CRF_01AE/B/C (0.3%, Figure 1J). The prevalence of URF (18.4%) was similar to that of CRF01_AE (17.7%) and revealed frequent recombination among the main strains (subtype C, CRF01_AE and subtype B). Most URFs (76.4%, 42/55) were found in individuals infected through heterosexual contact (Table 2). Seven of the HIV sequences derived from the 10 Burmese individuals were subtype C and the other 3 were URF_BC.

**Table 2:** The constitution of HIV-1 subtypes in different transmission routes

**Transmission route**	**Subjects**	**C**	**URFs**	**CRF01_AE**	**B**	**CRF08_BC**	**CRF07_BC**	**χ**^**2**^	P*
Heterosexual	227 (100%)	89 (39.2%)	42 (18.5%)	43 (18.9%)	25 (11.0%)	24 (10.6%)	4 (1.8%)	13.801	0.013
Intravenous drug injection	52 (100%)	32 (61.5%)	9 (17.3%)	3 (5.8%)	7 (13.5%)	1 (1.9%)	0 (0%)		
Homosexual	2 (100%)	0 (0%)	0 (0%)	1 (50.0%)	0 (0%)	0 (0%)	1 (50.0%)		
Mother to child	4 (100%)	1 (25.0%)	1 (25.0%)	2 (50.0%)	0 (0%)	0 (0%)	0 (0%)		
Unknown	14 (100%)	7 (50.0%)	3 (21.4%)	4 (28.6%)	0 (0%)	0 (0%)	0 (0%)		

*: Compared the proportions of subtypes between groups of heterosexual contact and intravenous drug injection.

The presence of subtypes/CRFs varied with different transmission routes. As shown in Table 2, all 6 genotypes were found in the heterosexually transmitted population. Five genotypes, with the exception of CRF07_BC, were detected in IDUs. One subtype C, 1 URF and 2 CRF01_AE strains were detected in the 4 young children infected through mother-to-child transmission; 1 CRF01_AE strain and 1 CRF07_BC strain in 2 persons who were men having sex with men. The proportions of HIV-1 genotypes in heterosexually transmitted population and IDUs showed a statistical difference (χ^2^=13.801, *p*=0.013) suggesting that different subtype distribution patterns were associated with different infection routes. The proportion of subtype C among IDUs (61.5%) was significantly higher than that among the heterosexually transmitted population (39.2%, χ^2^=8.591, *p*=0.005). Likewise, the proportion of CRF01_AE in the heterosexually transmitted population (18.9%) was significantly higher than that among IDUs (5.8%, χ^2^=5.332, *p*=0.022). The proportions of URFs, subtype B and CRF08_BC showed no statistical difference in these two populations.

### Genotypic analysis of DR

To detect TDR-associated mutations, the *pol* sequences were screened against those within the Stanford HIV Drug Resistance Database (http://hivdb.stanford.edu). DR-related mutations were identified in 13 of the 299 viral sequences (4.3%). With the exception of one individual who had 2 NNRTI mutations, the other 12 individual harbored virus with 1 DR mutation each. The demographic characteristics of these 13 ART-naive individuals are shown in Table 3. The mean age was 32.5 years. The mean CD4 cell count was 467 cells/μl and 4 individuals were ART eligible. No statistical difference was observed in distribution of DR strains by gender, age, infection route, ethnicity, CD4 cell count or HIV-1 subtype. Eight were infected through heterosexual transmission and the other 5 through intravenous drug injection. One individual (YN10S0137) was Burmese.

**Table 3:** Demographic characteristics of 13 individuals infected by viruses containing DR mutations

**Sequence ID**	**Gender**	**Age**	**Transmission route**	**Ethnicity/Nationality**	**CD4**^**+**^**Count/μl**	**DR Mutation**			**HIV-1 Subtype**
						**NRTI**	**NNRTI**	**PI**	
YN09S0004	Male	37	Intravenous drug injection	Jingpo	383	-*	K103N	-	B
YN09S0012	Male	55	Heterosexual	Han	506	-	-	M46I	BC
YN09S0021	Female	42	Heterosexual	Jingpo	149	-	K103N	-	CRF01_AE
YN09S0027	Female	29	Heterosexual	Achang	225	-	K101E	-	C
YN09S0054	Female	38	Heterosexual	Jingpo	280	-	K103N, Y181C	-	BC
YN09S0072	Female	31	Heterosexual	Jingpo	422	-	K103NS	-	B
YN09S0092	Female	19	Intravenous drug injection	Han	335	-	Y181C	-	C
YN09S0114	Male	24	Heterosexual	Jingpo	882	-	G190A	-	C
YN09S0129	Male	25	Heterosexual	Jingpo	608	-	-	L90M	CRF08_BC
YN10S0097	Male	34	Intravenous drug injection	Han	639	-	-	I85V	C
YN10S0132	Male	33	Intravenous drug injection	Dai	475	-	Y181C	-	C
YN10S0137	Female	22	Heterosexual	Burmese	473	M184I	-	-	C
YN10S0138	Male	33	Intravenous drug injection	Jingpo	687	-	-	I85V	C

*: not detected.

The proportion of sequences with TDR mutation associated with NRTIs, NNRTIs, and PIs was 0.3% (n=1), 2.7% (n=8), and 1.3% (n=4), respectively. Sequences containing mutations associated with 2 or 3 classes of antiretroviral drugs were not found. The PI-related DR included: I85V (0.67%, n=2), M46I (0.33%, n=1) and L90M (0.33%, n= 1). Although I85V is a nonpolymorphic PI-selected mutation, it does not result in resistance to PI. Only 1 NRTI-related DR mutation, M184I (0.33%, n=1), was detected. Among NNRTI-related DR mutations, K103N and Y181C were found in 3 samples (1.0%). K103N causes high-level resistance to nevirapine (NVP) and efavirenz (EFV). Y181C causes high-level resistance to NVP and intermediate resistance to EFV, etravirine **(**ETR) and rilpivirine **(**RPV). Importantly, Y181C forms the foundation for high-level ETR and RPV resistance as an addition of some single mutations and many double mutations causes high-level resistance to these drugs. Three individuals were found to have one other NNRTI-related mutation (K103NS, K101E and G190A) each (0.33%).

**Figure 2:**
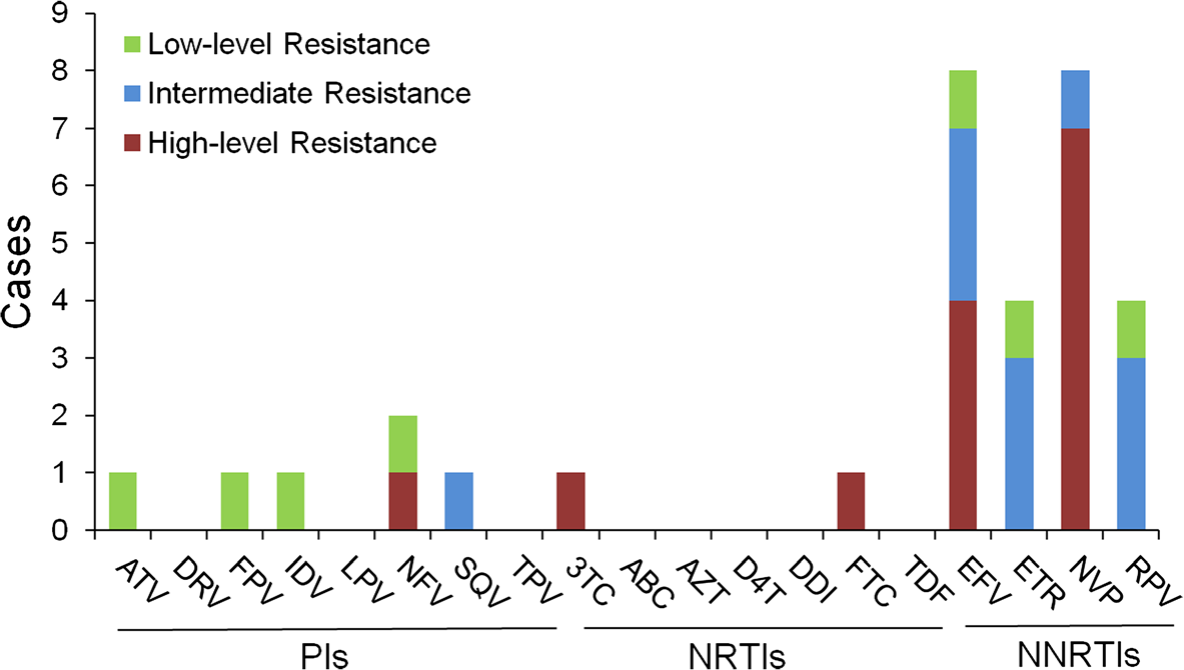
**The presence of DR from 13 individuals with DR strains.** PI: protease inhibitor; NRTI: nucleoside reverse transcriptase inhibitor; NNRTI: non-nucleoside reverse transcriptase inhibitor; ATV: atazanavir; DRV: darunavir; FPV: fosamprenavir; IDV: indinavir; LPV: lopinavir; NFV: nelfinavir; SQV: saquinavir; TPV: tipranavir/r; 3TC: lamivudine;ABC: abacavir;AZT: zidovudine;D4T: stavudine;DDI: didanosine;FTC: emtricitabine;TDF: tenofovir;EFV:efavirenz; ETR: etravirine;NVP:nevirapine;RPV:rilpivirine.

Based on the interpretation of the Stanford HIV Drug Resistance Database, the 13 drug resistant viruses showed different levels of resistance to 11 antiretroviral drugs (Figure 2). Although lopinavir (LPV) and indinavir (IDV) were the main PIs used in Dehong, low-level resistance to atazanavir (ATV), fosamprenavir (FPV), IDV, nelfinavir (NFV) and saquinavir (SQV) was detected. Among RTIs, intermediate and high-level resistance to 4 NNRTIs (EFV, ETR, NVP and RPV) and high-level resistance to 2 NRTIs (lamivudine (3TC) and emtricitabine (FTC)) were detected, of which 2 NNRTIs (ETR and RPV) and 1 NRTI (FTC) were not used in local patients. The reason for their presence might be due to cross-resistance, for example 3TC induced M184I/V but also rendered resistance to FTC. Further, cross-resistance was a common phenomenon among NNRTIs. The existence of cross-resistance could increase the complexity and the length of ART.

## Discussion

Yunnan has been described as a “key HIV epicenter in China” [21]. By the end of 2010, the cumulative number of HIV/AIDS cases reported in Yunnan was 83,925 and accounted for 21% of total HIV/AIDS cases in China. With the scale up of ART in Yunnan, the emergence of HIV DR is anticipated. The surveillance of emergence and transmission of drug resistant HIV-1 strains is one of the priorities for HIV/AIDS prevention and control in Yunnan. In particular, surveying the characteristics and trends of TDR among newly reported cases of HIV infection could provide valuable information for evaluating the efficacy of ART, public health planning [22], and the selection of pre- and post-exposure prophylaxis.

By using a standard genotyping protocol, we carried out a survey of TDR in 299 newly HIV-1 diagnosed and ART-naive individuals residing in Dehong. Our study revealed a low TDR rate of 4.3%. An earlier study conducted in 2006 of 64 newly infected HIV-1 patients whose infections were classified to be recent by BED incidence assay yielded a rate of 6.3% [23]. No statistical difference between the rates of these 2 studies (*p*>0.05) was found. This indicates that the prevalence of drug resistant HIV-1 strains remained relatively constant over the 5 years between 2006 and 2010, and that the management of treated individuals with standardized ART protocol appeared to be efficient in western Yunnan. In this study, we also found one DR strain among 10 Burmese individuals. Further vigilance needs to be placed to monitor the importation of DR strains from neighboring countries.

The first-line regimens of the Chinese national free ART program include 2 NRTIs and 1 NNRTI. The ART drugs presently used in Dehong include 6 NRTIs (abacavir (ABC), zidovudine (AZT), stavudine (D4T), didanosine (DDI), tenofovir (TDF) and 3TC, two NNRTIs (EFV and NVP), and two PIs (LPV and IDV). A total of 69.2% of drug resistant strains found in this study were RTI-associated. These DR mutations (M184I, K103N/S, Y181C and K101E) could derive from ART-treated patients in Yunnan [24]. Further, intermediate and high-level resistance to four NNRTIs was detected with increased frequency. The increased rates of RTI relevant resistance strains could increase the failure of the first-line antiretroviral regimens. The use of PIs is limited and PI-related DR mutations were not previously found among the treated population in 2008 in Yunnan [24]. Five PI-related DR strains were detected in this study. A possible explanation for the presence of DR to PIs different from the ones used in Dehong (LPV and IDV) is that the individuals were administering self-procured PIs from overseas or the observed mutations were introduced by foreigners.

In this study, we found that most subtypes/CRFs were present in both the heterosexually transmitted population and IDUs. This finding suggests that various HIV-1 subtypes/CRFs were disseminating between different high-risk populations via complicated transmission routes and/or introduced by bridging populations such as female sex workers. Extensive BC recombinants were previously detected among IDUs in Dehong [9,10,12]. In this work, we found increased intersubtype recombinants in IDUs and the heterosexually transmitted population. In addition to BC recombinants, CRF01_AE and subtype C or BC recombinants were also identified for the first time in this area. Recently, extensive and complex HIV-1 recombination events between B', C and CRF01_AE were found in the neighboring northern Myanmar area [25]. Thus, the China-Myanmar border area appears to be a hot spot of active viral recombinant events. In order to effectively control the HIV epidemic in this area, comprehensive strategies including risk behavior interventions, enhanced HIV testing and proper use of ART should be emphasized.

As a linkage between the HIV-1 epidemics in Southeast Asia and China, Dehong converges many HIV-1 strains circulating in this area [8,13,26,27]. Dehong was also affected by the epidemic in other parts of China. For example, CRF07_BC and CRF08_BC were first detected from IDUs in Xinjiang province and Guangxi province in 1997, respectively [28,29]. However, it is believed that these two CRFs were initially formed in Yunnan Province [30] and spread through two different overland heroin trafficking routes. CRF07_BC spread northwestward to Xinjiang, and CRF08_BC eastward to Guangxi. As reported previously, these 2 CRFs were not identified in western Yunnan until 2002 [9,10,12] but were predominant in eastern Yunnan prior to 2002, including Honghe and Wenshan prefectures [12]. Thus, it is likely that these 2 CRFs might have formed in eastern Yunnan and spread to western Yunnan. Furthermore, the epidemic could potentially influence neighboring countries if infected individuals and drug trafficking are not monitored and managed efficiently.

## Conclusions

This study of HIV-1 genetic diversity and TDR-associated mutations among ART-naive individuals in Dehong, Yunnan reveals distinct subtype distribution patterns in the heterosexually transmitted population and IDUs. While subtype C was the most frequently found virus in both populations, CRF01_AE was found to circulate more frequently in the heterosexually transmitted population. Strikingly, the URF recombinant events continued to rise with a prevalence second only to subtype C. The overall prevalence of TDR remained low in newly HIV-1 diagnosed and ART-naive individuals. HIV drug resistant mutations in these individuals represent a great challenge for the future of the ART program. Our findings could better guide us in the development of efficacious and effective management plans and strategies to prevent the transmission of drug resistant strains of HIV-1.

## Abbreviations

DR: Drug Resistance; TDR: Transmitted Drug Resistance; ART: Antiretroviral Therapy; IDU: Injecting Drug User; PI: Protease Inhibitor; NRTI: Nucleoside Reverse Transcriptase Inhibitor; NNRTI: Non-Nucleoside Reverse Transcriptase Inhibitor; PR: Protease; RT: Reverse Transcriptase; URF: Unique Recombinant Form; CRF: Circulating Recombinant Form.

## Competing interests

The authors declare that they have no competing interest.

## Authors’ contributions

LL and MJ for project initiation; MC, YM, SD, SY, YS, HL, LY and HC for data collection; MC, YM and HX for data analysis; MC, YM, AQ, LL, MJ and CYO for manuscript preparation. All authors read and approved the final manuscript.

## Pre-publication history

The pre-publication history for this paper can be accessed here:

http://www.biomedcentral.com/1471-2334/12/382/prepub
